# A Deep Ultraviolet Mode-locked Laser Based on a Neural Network

**DOI:** 10.1038/s41598-019-56845-6

**Published:** 2020-01-10

**Authors:** Haoyuan Lu, Hao Xu, Jianye Zhao, Dong Hou

**Affiliations:** 1 Department of Electronics, Peking University, No.5 Yiheyuan Road, Haidian District, Beijing, 100871 China; 2 School of Automation Engineering, University of Electronic Science and Technology of China, Chengdu, Sichuan 611731 China

**Keywords:** Computer science, Transformation optics

## Abstract

Deep ultraviolet lasers based on the phenomenon of mode-locking have been used widely in many areas in recent years, for example, in semiconductors, the environment and biomedicine. In the development of a mode-locked deep ultraviolet laser, one of the most important aspects is to optimize the multiple parameters of the complex system. Traditional optimization methods require experimenters with more optimization experience, which limits the wide application of the lasers. In this study, we optimize the deep ultraviolet mode-locked laser system using an online neural network to solve this problem. The neural network helps us control the position of the crystal, the length of the cavity, the position of the focusing lens and the temperature of the frequency doubling crystal. We generate a deep ultraviolet mode-locked laser with a power of 18 mW and a spectral center at 205 nm. This result is greatly improved compared to previous results with the same pump power. This technology provides a universal solution to multiparameter problems in the optimization of lasers.

## Introduction

Deep ultraviolet (DUV) lasers have many practical applications in many fields. Almost all materials have a spectral fingerprint in the ultraviolet range^1–8^ (especially at 30–200 nm). With increasing attention on deep ultraviolet ellipsometry and more research on the interactions between DUV lasers and matter, the application of deep ultraviolet spectroscopy has spread from various fields of biomedicine^9–12^ to the creation of novel functional materials^13–17^. Also many important transitions, such as the Hydrogen 1S-2S two-photon transition^18–20^ (243 nm), have DUV and shorter wavelengths. The DUV laser is a promising tool for learning about C, H, N, and O atoms in organic chemistry^21^.

However, it is almost impossible to generate a DUV laser with sufficient power using a continuous wave laser. The frequency doubling of a mode-locked laser (MLL) is a viable way to obtain a stable DUV laser. For higher instantaneous powers and conversion efficiencies of the Ti:sapphire MLL, the lasers can convert IR lasers into the DUV region with nonlinear crystals. In addition, direct frequency comb spectroscopy (DFCS)^22–27^ with mode-locked lasers can extend spectroscopy into the DUV region.

Despite the significant advantages of optical combs over CW lasers, the DUV MLL is a more complex system with a set of experimental parameters. Changing one of these parameters will greatly affect the power, the wavelength and the center frequency of the frequency comb. In previous research, the experimenter optimized the parameters in the system based on their experience. Such parameter optimization always takes a lot of time to adjust the spectrum of the laser.

To avoid the parameter adjustment problem, a fiber laser is often used in the conventional mode-locked-laser scheme^28^. The fiber mode-locked laser is stable, and fiber laser parameter optimization is simpler. However, it is almost impossible to generate a fiber mode-locked laser with sufficient power and a high repletion rate in the DUV region. Therefore, we optimize a Ti:Sapphire laser to generate the DUV laser. Compared with the fiber laser, if we can solve the parameter optimization problem, the Ti:Sapphire laser can generate sufficient power easily. It also has a lager adjustable laser spectral range and low phase noise.

At the same time, many recent studies^29–34^ have used neural networks to solve the multiparameter adjustment problem of complex systems in experiments and have shown great advantages over human. Here, these techniques are utilized in lasers and related applications, such as laser cutting^35^, optical communication^36,37^ and mode-locked laser^38^. While these techniques effectively improve the performance of lasers, these technologies are based on a priori information of offline optimization. This means that these techniques are difficult to optimize in real time for environmental changes that occur while the experiment is taking place.

In this study, we optimize the DUV MLL system using an online neural network to obtain the target spectrum of lasers and improve the power of the laser. There are some differences between DUV ultrafast laser optimization and other parameter optimization problems. First, the DUV ultrafast laser is extremely sensitive to changes in various environments. The parameters need to be adjusted in real time to cope with slow changes in the experimental environment. Second, the DUV ultrafast system is difficult to move. Therefore, it is difficult to collect enough data from the system in different environments. Other optimization technologies need large datasets. Thus, we choose the Artificial neuron network (ANN) as our optimization tool. Our ANN can perform real-time operations to give the optimal parameter solution in the current environment. We train the ANN each time because it can be trained quickly based on the current environment. When the spectrum and power meet the requirements, the laser can be used for deep ultraviolet direct frequency comb spectroscopy detection of the Hydrogen 1S-3S two-photon transition.

## Results

### Neural network for optimization design

**Figure 1:**
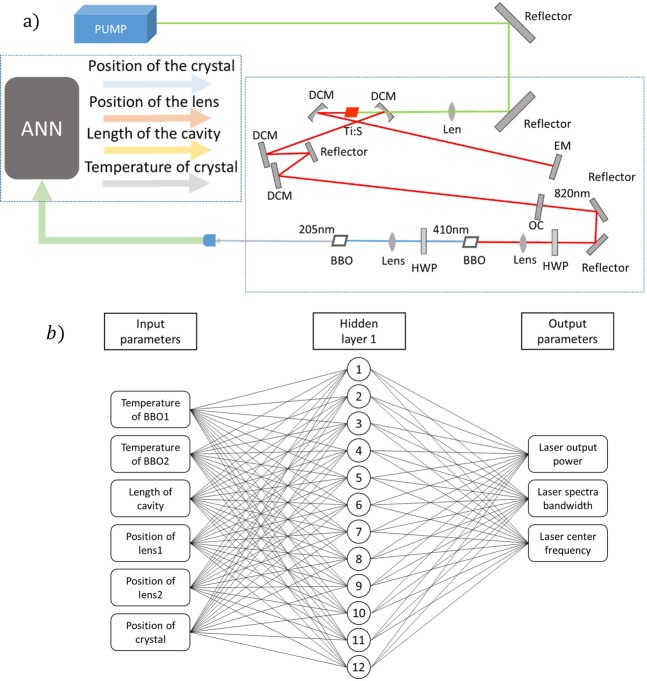
(**a**) Simplified block diagram of the system. DCM: double-chirped mirror; EM: end mirror; Ti:S: Ti:Sapphire crystal; OC: output coupler; BBO: BaB_2_O_4_ crystal; HWP: half-wave plate; ANN: artificial neural network. (**b**) Structure of artificial neural network.

The DUV MLL system is shown in Fig. 1a.

In the experiment, we found that the position of the crystal, the length of the cavity, the position of the focusing lenses and the temperature of the frequency doubling crystals are the parameters that have the greatest influence on the laser output power and spectrum. We need to constantly change several of these parameters to optimize the results. However, tuning any of these parameters requires changes to other parameters at the same time, which greatly increases the optimization difficulty. Therefore, in the next experiment, we used stepping motor stages to adjust the position of the crystal mold, the cavity mirror, and the lenses, and we used Thermo Electric Coolers (TECs) to adjust the crystal temperature. This facilitates the introduction of a neural network to improve the output of the laser.

To improve the laser output, we introduced the ANN optimization system. The ANN structure is shown in Fig. 1b. We divide the position variables controlled by 4 stages, each of which is divided into 25 bins each. The temperature variables controlled by the two TECs are divided into 20 bins. The neural network designed in this study is a feed-forward network with a single hidden layer and one output layer. The single-layer network ensures fast operation for real-time optimization. Based on ref. ^34^,it has been demonstrated by proof that networks with a single hidden layer that have a sufficient number of neurons are universal approximators. The number of nodes in the hidden layer is 12. The three node considered in the output layer are the output power, the laser bandwidth and the laser center frequency.

We choose the Gaussian error linear unit (GELU) as the activation function. We simply evaluate the activation function with the laser output power after optimization. The laser output power with the GELUs is 18.43 mW. The laser output power with a sigmoid function is 15.2 mW. The laser output power with a Rectified Linear Unit is 16.75 mW. We plan to evaluate the activation function with both the output power and the line width in future. We choose the genetic algorithm for the training algorithm, which was verified to be very effective in our subsequent experiments. Genetic algorithm can help network find a global optimum point. We use open source machine learning algorithms provided by Ref. ^29^.

We tried to use multiple ANNs to optimize the results, but it seems that the effect is not very good in the reform experiment. Multiple ANNs can ensure that the ANN does not become confined in local minima. However, it has been found in experiments that such problems are rare in laser power and laser spectral width. Multiple ANNs will make the network run time much longer. This makes our real-time optimization goals difficult to achieve. To speed up the optimization, we only use one ANN for optimization. Because the network topology is relatively simple, parameter adjustment can be done on an ordinary home computer. The processor of our computer is an Intel i7-6700k@4.0 GHz.

The conjugate gradient algorithm is utilized to predict the parameter values for minimizing the cost function. The laser parameters are changed by the predicted values. Then, we measure the laser outputs of energy and spectrum and update the weight of each neuron according to the results. Repeat this process until the function converges for training. However, slow change of environment will still influence the laser output after the function converges. For real-time optimization, we always keep the neural network running without genetic algorithm after function converges. The neural network completes a run in one second and updates the parameters in real time based on the results. This results in long-term stable operation of the DUV laser.

### Optimization results

**Figure 2:**
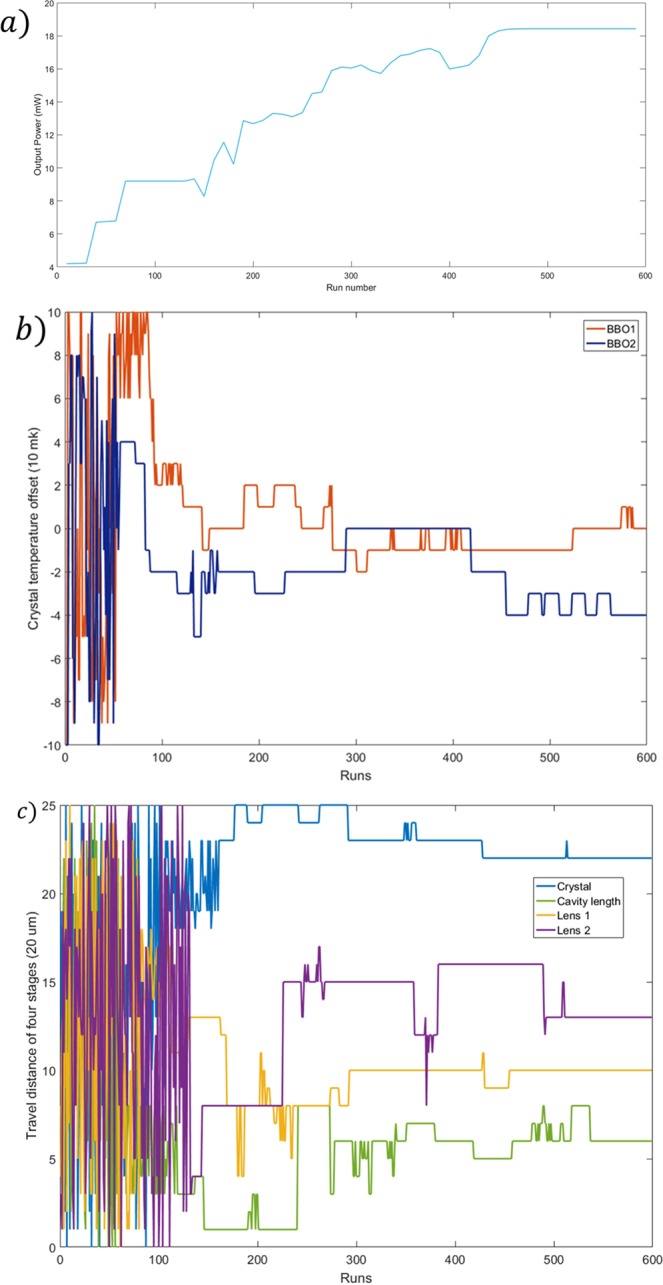
Optimization process of laser power. (**a**) Optimization results of laser power. (**b**) Travel distance of four stages, where the position of the crystal, the length of the cavity, and the position of the focusing lenses are set by the ANN. (**c**) Crystal temperature set by the ANN.

Our subsequent experimental plan is to use the optimized laser to excite the 1S-3S two-photon transition line of hydrogen atoms. For this goal, we will use neural networks to optimize the laser output power and spectrum. The optimization of laser power is relatively simple. The cost function can be given directly by the laser power. The laser output power can be tested by means of a power meter. By bringing the cost function directly into the neural network established above, we can optimize the DUV laser output power. Due to the limitation of the response time of the UV power meter, each run of the neural network optimization takes 1 s. The optimization results are shown in Fig. 2. We can see that after approximately 500 runs, the laser output power converges to 18.43 mW. The laser output power fluctuates within 0.2 mW within one day. At this time, the measured pulse width is 350 fs. This result is better than the previous maximum laser output power of 11 mW, and the optimization time can be controlled within 15 min, which has a greater advantage than manual optimization. However, we noticed that the laser output angle will change with the change of the stage, and the power meter receiving efficiency will also change slightly. This phenomenon will interfere with the optimization of the laser power. If we choose the pulse width as the node in the output layer, we can reduce the pulse width to 152 fs. However, output power is approximately 13 mW.

**Figure 3:**
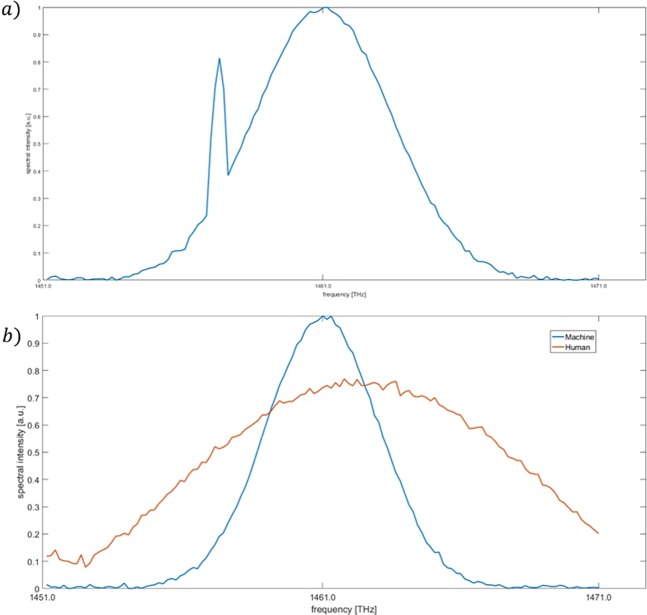
(**a**) Optimized spectrum when we built the cost function directly from the difference between the measured laser center and the set target value. (**b**) Comparison between the human-optimized spectrum and the machine-optimized spectrum.

Of course, for deep UV DFCS detection, laser spectroscopy is a more important indicator than laser energy. The attachment power of the deep ultraviolet laser at the target transition directly determines the spectral signal-to-noise ratio and other indicators. However, the cost function of laser spectroscopy is more complex than laser power. We initially built the cost function directly from the difference between the measured laser center and the target value we set, but the effect is not ideal. In Fig. 3a, we use the output power as the cost function. This is the neural network optimized only for laser power. After optimization, the spectrum is not flat, and a large DC component appears. At this time, the spectrum is often not satisfactory to us. Under such parameters, the pump optical power is too large for the laser to cause the spectrum to produce a DC component. The DC component is useless for TPT excitation. Thus, the energy at the center of the laser spectrum is not the best. In addition, for the detection of DFCS, the modes at the transition line can excite the atomic two-photon transition, but the modes on either side of the transition line can also excite atomic transitions in pairs. The excitation probability of different modes is s, as given in Ref. ^39^

1 $${{\rm{P}}}_{k\to m}=\sum _{n{\rm{^{\prime} }}}\frac{{E}_{0}^{4}|\langle {u}_{m}|D|{u}_{n{\rm{^{\prime} }}}\rangle \langle {u}_{n{\rm{^{\prime} }}}|D|{u}_{k}\rangle {|}^{2}}{4\hslash ({\omega }_{nk}-{\omega }_{n{\rm{^{\prime} }}k})}\frac{si{n}^{2}[({\omega }_{mk}-{\omega }_{n{\rm{^{\prime} }}k}-{\omega }_{m{\rm{^{\prime} }}n{\rm{^{\prime} }}})t]}{{({\omega }_{mk}-{\omega }_{n{\rm{^{\prime} }}k}-{\omega }_{m{\rm{^{\prime} }}n{\rm{^{\prime} }}})}^{2}}$$

where the ground state is k, the middle state is n, and the excited state is m. ${\omega }_{nk}$ and ${\omega }_{m^{\prime} n^{\prime} }$ are the frequencies of the two photons.

Thus, we changed the evaluation of the laser spectrum. We use a spectrometer to detect the laser spectrum, calculate the energy of 1 THZ around the transition line through the spectrum, and assign weights according to the excitation probability of the two-photon transitions in different frequency ranges. Each optimization takes 5 s because it is limited by the spectrometer detection speed. The optimization time is within 30 minutes. Figure 3b shows an optimized laser spectrum, and its excitation line intensity will be tripled by manual optimization according to Eq. 1. Comparing the results of Fig. 3b with those of Fig. 3a, the laser output energy is similar after replacing the cost function, but it has more energy near the target frequency.

## Discussion

In this study, we successfully used neural networks to optimize our DUV mode-locked lasers. We generate a DUV laser with a power of 18 mW and a spectral center at 205 nm. This result has been greatly improved compared to the previous result with the same pump power. The optimized laser can be utilized to detect the atomic spectrum and measure the hyperfine constants through the spectrum. We now save run time for real-time optimization, simplifying the setup of the entire network. In future work, we plan to use a more powerful device to run the network. We can use more hidden layers in the network and multiple ANNs. This can increase the output power. In addition, according to Ref. ^27^, the LBO crystal is more suitable for the first frequency doubling stage. It can also increase the output power.

Of course, this technology is not limited to DUV mode-locked lasers, and it can also be utilized to solve multiparameter adjustment problems in other solid-state lasers. The multiparameter optimization problem mentioned above is very common and is difficult to avoid. The introduction of neural networks seems to provide a universal solution to this type of problem. It can help a large number of experimenters who have no experience in optimizing lasers. This technology will have broad application prospects.

## Methods

### Ti:sapphire laser

The laser cavity is based on a four mirror X-folded resonator. The laser cavity has a 3-mm-long Ti:sapphire crystal. The position of the crystal can be adjusted by a stepping motor stage. The radius of curvature (ROC) of the folding mirrors is 75 mm. All mirrors in the cavity are double-chirped mirrors (DCM), which are GDD-oscillation compensated in pairs. The mode-locked laser emits 20-fs pulses with an average power of 800 mW and a repletion rate of 100 MHz. The cavity length can be adjusted via a PZT and a stepping motor stage in the cavity.

### Frequency quadruple

We focus the beam into two BaB_2_O_4_ (BBO) crystals by lenses to frequency quadruple the laser. The crystals are Brewster cut. The first BBO crystal is used to adjust the frequency of the laser from 820 nm to 410 nm. The crystal is 8 mm long with θ = 90° and φ = 28.5° for type I phase matching (820 nm (o) → 410 nm (e)). The temperature of the crystal is controlled to 40 °C. We will change the polarization of the 410-nm laser with a half-wave plate and focus it into the other crystal. The BBO crystal is used for the second doubling stage. The crystal is 8 mm long with θ = 86.4° for type I phase matching (410 nm (o) → 205 nm (e)). The temperature of the crystal is controlled to −10 °C. Finally, we can get a 205-nm mode-locked laser.
